# The International Medical Annual and Practitioner’s Index for 1893

**Published:** 1893-05

**Authors:** 


The International Medical Annual and Practitioner's Index for 1893. Edited by a corps of thirty-eight department editors—European and American—specialists in their several departments. P. W. Williams, M. D., Secretary of Staff. Six hundred and twenty-six octavo pages. Illustrated. $2.75. E. B. Treat, Publisher, 5 Cooper Union, New York.

The eleventh yearly issue of this valuable one-volume reference work is to hand; and it richly deserves and perpetuates the enviable reputation which its predecessors have made, for selection of material, accuracy of statement, and great usefulness. The corps of department editors is representative in every respect. Numerous illustrations—many of which are in colors—make the "Annual" more than ever welcome to the profession, as providing, at a reasonable outlay, the handiest and best *résumé* of Medical Progress yet offered.

Part I comprises the new remedies, together with an extended review of the therapeutic progress of the year.

Part II, comprising the major portion of the book, is given to the consideration of new treatment; and is a retrospect of the year's work, with several original articles by eminent authorities.

The third—and last part—is made up of miscellaneous articles, such as Recent Advances in Sanitary Science;. Improvements in Pharmacy; New Inventions in Instruments and Appliances; Books of the Year, etc.

The arrangement of the work is alphabetical, and with its complete Index, makes it a reference book of rare worth.

In short, the "Annual" is what it claims to be—a recapitulation of the year's progress in medicine, serving to keep the practitioner abreast of the times with reference to the medical literature of the world. Price, the same as in previous years, $2.75.

